# Psilocybin as a New Approach to Treat Depression and Anxiety in the Context of Life-Threatening Diseases—A Systematic Review and Meta-Analysis of Clinical Trials

**DOI:** 10.3390/biomedicines8090331

**Published:** 2020-09-05

**Authors:** Ana Sofia Vargas, Ângelo Luís, Mário Barroso, Eugenia Gallardo, Luísa Pereira

**Affiliations:** 1 Centro de Investigação em Ciências da Saúde (CICS-UBI), Universidade da Beira Interior, Av. Infante D. Henrique, 6200-506 Covilhã, Portugal; anadinisvargas@gmail.com (A.S.V.); angelo.luis@ubi.pt (Â.L.); egallardo@fcsaude.ubi.pt (E.G.); 2 Grupo de Revisões Sistemáticas (GRUBI), Faculdade de Ciências da Saúde, Universidade da Beira Interior, Av. Infante D. Henrique, 6200-506 Covilhã, Portugal; 3 Laboratório de Fármaco-Toxicologia, UBIMedical, Universidade da Beira Interior, Estrada Municipal 506, 6200-284 Covilhã, Portugal; 4 Serviço de Química e Toxicologia Forenses, Instituto Nacional de Medicina Legal e Ciências Forenses, I.P.—Delegação do Sul, Rua Manuel Bento de Sousa, 3, 1150-219 Lisboa, Portugal; mario.j.barroso@inmlcf.mj.pt; 5 Centro de Matemática e Aplicações (CMA-UBI), Universidade da Beira Interior, Rua Marquês d’Ávila e Bolama, 6201-001 Covilhã, Portugal

**Keywords:** psilocybin, depression, anxiety, clinical trials, systematic review, meta-analysis

## Abstract

Psilocybin is a naturally occurring tryptamine known for its psychedelic properties. Recent research indicates that psilocybin may constitute a valid approach to treat depression and anxiety associated to life-threatening diseases. The aim of this work was to perform a systematic review with meta-analysis of clinical trials to assess the therapeutic effects and safety of psilocybin on those medical conditions. The Beck Depression Inventory (BDI) was used to measure the effects in depression and the State-Trait Anxiety Inventory (STAI) was used to measure the effects in anxiety. For BDI, 11 effect sizes were considered (92 patients) and the intervention group was significantly favored (WMD = −4.589; 95% CI = −4.207 to −0.971; *p*-value = 0.002). For STAI-Trait, 11 effect sizes were considered (92 patients), being the intervention group significantly favored when compared to the control group (WMD = −5.906; 95% CI = −7.852 to −3.960; *p*-value ˂ 0.001). For STAI-State, 9 effect sizes were considered (41 patients) and the intervention group was significantly favored (WMD = −6.032; 95% CI = −8.900 to −3.164; *p*-value ˂ 0.001). The obtained results are promising and emphasize the importance of psilocybin translational research in the management of symptoms of depression and anxiety, since the compound may be effective in reducing symptoms of depression and anxiety in conditions that are either resistant to conventional pharmacotherapy or for which pharmacologic treatment is not yet approved. Moreover, it may be also relevant for first-line treatment, given its safety.

## 1. Introduction

Major depressive disorder (MDD) is characterized by the persistence of negative thoughts and emotions that disrupt mood, cognition, motivation, and behavior [1]. According to the Diagnostic Statistical Manual V (DSM), symptoms, etiologies and pathophysiologies of MDD are heterogeneous and their subtypes have been already described. Although symptomatic remission increases the probability for recovery in MDD, most patients do not achieve nor sustain a state of full remission [2]. The options for treatment when the patient is resistant to the available standard treatments generally involve combining, augmenting, or switching medications, introducing electroconvulsive therapy (ECT) or other neurostimulation strategies. However, the risk of complications associated with those approaches exists, including increased toxicity with higher medication dosages and combination regimens [1].

Anxiety is a very common psychiatric symptom in terminally ill patients. Factors such as treatment process, disease progression, uncontrolled pain, dying and uncertainty about death have a negative impact on these patients [3]. Anxiety also contributes to poor recovery from medical procedures and lower survival time in terminally-ill patients [4].

Depression and anxiety are independent risk factors of early death in patients with life-threatening diseases, like cancer [5] and most of these individuals develop a chronic syndrome of psychological distress, usually associated with decreased treatment adherence, prolonged hospitalization, decreased quality of life and increased suicidality [6]. Antidepressants and benzodiazepines are used to treat depressed mood and anxiety in patients with life-threatening diseases [7], although there are no Food and Drug Administration (FDA) approved pharmacotherapies for the psychological distress related to those diseases. In addition, the onset of clinical improvement with antidepressants is delayed, relapse rates are high and significant side effects compromise adherence to therapy [8].

Regarding the treatment of severe depression and anxiety, special attention should be paid to the approval of intranasal Spravato^®^ by the FDA in March 2019. Its active compound is esketamine, a ketamine enantiomer, which is a non-competitive *N*-methyl-d-aspartate (NMDA) glutamate receptor antagonist. The effects of ketamine have made it a popular recreational drug, due to its euphoric and dissociative properties [9]. Nonetheless, esketamine appears to provide significant short-term symptom improvement in severe depression and anxiety and it is considered an innovative and promising therapeutic approach. Moreover, molecules such as 3,4-methylenedioxy-methamphetamine (MDMA) and lysergic acid diethylamide (LSD) are also being studied for the treatment of anxiety conditions, namely, post-traumatic stress disorder (PTSD) and generalized anxiety disorder (GAD). Nevertheless, none of these drugs has been approved by the FDA.

In spite of all studies and therapeutic innovations in this area, further research is needed, notably on classic hallucinogens like psilocybin, to provide patients with depression and anxiety associated with life-threatening diseases better chances of recovery and consequently better quality of life.

Recently, Goldberg et al., 2020 [10] published a meta-analysis concerning the use of psilocybin and symptoms of anxiety and depression. The approach used in their meta-analysis is different from the one we present in several aspects, for instance—it does not include data on previous pathologies of the patients receiving psilocybin and did not analyze physiological effects induced by the drug. Also, the authors did not examine psilocybin dose and administration duration as moderators of treatment effects [10].

In this context, this paper aims to perform a systematic review, complying with the Preferred Reported Items for Systematic Reviews and Meta-Analysis (PRISMA) statement, followed by a meta-analysis of clinical trials on the therapeutic potential of psilocybin in anxiety and depression associated with life-threatening diseases.

## 2. Methods

### 2.1. Search Strategy, Study Selection, Inclusion and Exclusion Criteria

The search for this systematic review with meta-analysis was performed on several electronic databases (PubMed, Web of Science, Scopus and SciELO) during January 2020. These databases were queried using the Boolean operator tools with the following search strategy—((((((depression) OR anxiety) OR post-traumatic stress disorder)) AND ((((((hallucinogens) OR entheogens) OR psychedelics) OR classic hallucinogens) OR serotonergic hallucinogens) OR serotoninergic hallucinogens)) AND (psilocybin) AND ((((((clinical trials) OR human) OR humans) OR man) OR woman) OR patients). The references’ lists of the relevant studies were also checked to find further works. Following the PRISMA statement [11], titles and abstracts of the selected articles were initially screened and the full texts of those considered important were subsequently analyzed in detail [12]. The selection process of the studies was performed independently by two authors, with a third being consulted in case of disagreements. The defined inclusion criteria were—studies in humans presenting a true control group; studies in patients with depression and anxiety associated with a life-threatening disease; drug psilocybin; use of Beck Depression Inventory (BDI) to assess depression and/or State-Trait Anxiety Inventory (STAI) to assess anxiety.

BDI was first proposed by Beck et al., 1961 [13] and it is one of the most used scales to assess the severity of depressive symptoms. It is a self-reported questionnaire, consisting on 21 items that measure characteristic attitudes and symptoms of depression. STAI is a self-report questionnaire divided in 2 subscales, called STAI-Trait and STAI-State. STAI allows the detection of the presence and severity of current symptoms of anxiety and a generalized propensity to be anxious. It contains 40 items, 20 of them concerning STAI-State and the remaining 20 concerning STAI-Trait [14].

### 2.2. Risk of Bias Assessment

The risk of publication bias of each included study was assessed using the “Cochrane Guide for Review Authors on Assessing Study Quality”, which is based on “Cochrane Collaboration’s tool for assessing risk of bias” [15]. This tool classifies the risk of bias in randomized controlled trials (RCTs) included in reviews as either “High risk”, “Unclear” or “Low risk” in accordance with 7 domains—random sequence generation, allocation concealment, blinding of participants and personnel, blinding of outcome assessment, incomplete outcome data, selective reporting and other sources of bias [16]. This classification was independently assigned by 2 authors and discrepancies in assessment were resolved through discussions between the authors or by consultation with a third researcher. The results of the risk of bias assessment were presented in a risk of bias summary and a risk of bias graph, which were sketched using the software Review Manager 5.3 (Version 5.3.5) (http://community.cochrane.org/).

### 2.3. Data Extraction and Summary

The included studies were carefully analyzed, and the following data were extracted and summarized—publication year, sample size, study design, medical condition, age and gender of participants, conditions of intervention and control groups and study duration. The data extraction was independently performed by two authors using a prespecified procedure with a third reviewer consulted to analyze inconsistencies.

### 2.4. Statistical Analyses

For the outcomes of interest, an assessment was performed on the pooled effect of the treatment with psilocybin in terms of weighted mean differences (WMD) between the change from pre- and post-treatment mean values of the intervention and control groups. WMD combine measures, where the mean, standard deviation (SD) and sample size are known. The weight given to each study (how much influence each study has on the overall results of the meta-analysis) is determined by the precision of its estimate of effect and corresponds to the inverse of the variance. Statistical data analysis was performed using the Comprehensive Meta-Analysis software (Version 2.0) [17]. Forest plots were generated to illustrate the study-specific effect sizes with a 95% confidence interval (CI). The statistic I^2^ of Higgins was used as a measure of the inconsistency across the findings of the included studies. The scale of I^2^ has a range of 0 to 100% and values on the order of 25%, 50% and 75% are considered low, moderate, and high heterogeneity, respectively [18]. Subgroup analysis was performed on the primary outcomes, depending on the dose and on the follow-up time after psilocybin administration. Three different analyses were used to evaluate the potential impact of publication bias on the present meta-analysis—Funnel plots; Egger’s regression test; Duval and Tweedie’s Trim and Fill approach. The sensitivity analysis was also achieved by eliminating each study one at a time to evaluate the stability of the results.

## 3. Results

### 3.1. Search and Selection of Studies

**Figure 1:**
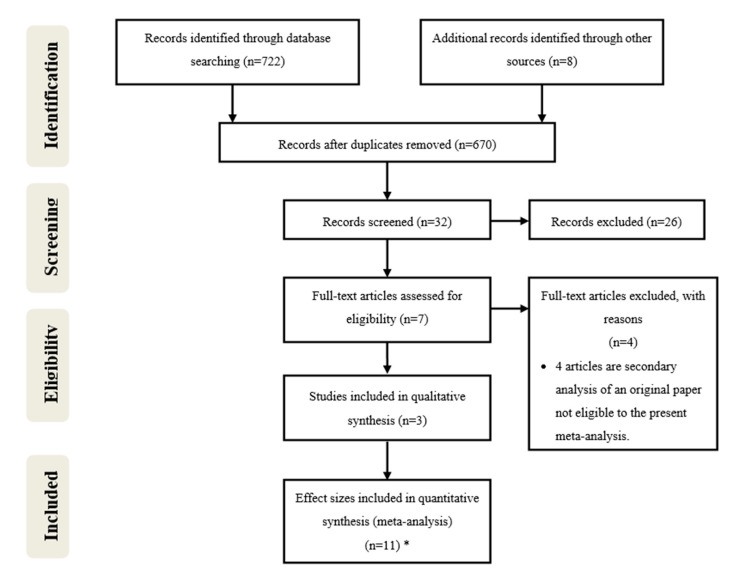
Preferred Reported Items for Systematic Reviews and Meta-Analysis (PRISMA) flow-diagram of database search, study selection and articles included in this systematic review with meta-analysis. * The works from Griffiths et al., 2016 [6], Ross et al., 2016 [19] and Grob et al., 2011 [20] were divided into several effect sizes based on different times of follow-up after psilocybin administration. (The division of each work in several effect sizes is indicated by the letters in unpaired parenthesis in Table 1).

The detailed steps of the article selection process are depicted as a flow-diagram (Figure 1). Initially, the search yielded 722 articles concerning the hypothetical therapeutic use of psilocybin. This initial search included 8 articles obtained through research in other sources. After, duplicates were removed, 670 articles remained and after the screening of titles and abstracts according to PRISMA statement, 32 of them remained and were further evaluated for inclusion and exclusion criteria. Among them, 26 did not meet the inclusion criteria.

Then, seven articles were assessed for eligibility through full-text evaluation and four of them were excluded (Stroud et al., 2018 [21], Lyons and Carhart-Harris, 2018 [22], Carhart-Harris et al., 2017 [23] and Carhart-Harris et al., 2016 [24]), because they were secondary analyses of the same data, and as such none was eligible since a true control group was not present in either of them. Finally, three articles were included in qualitative synthesis and three of them were divided into several effect sizes based on different times of follow-up after psilocybin administration. In a general view, the three articles studied the effects of psilocybin in the treatment of depression and anxiety associated with a life-threatening disease. The data from Ross et al., 2016 [19] was divided in seven data groups and the data from Griffiths et al., 2016 [6] and from Grob et al., 2011 [20] were divided into two data groups each, considering different times of follow-up after psilocybin administration. In total, 11 effect sizes were included in this meta-analysis.

### 3.2. Included Studies and Trials Characteristics

The main characteristics of the included trials are outlined in Table 1. The three included studies were published between 2011 and 2016 and 92 patients received psilocybin with doses ranging from 0.2 to 0.4 mg/kg, depending on the trials. The different units used to measure doses (some of them depending on the weight of the patients) made results standardization difficult. Patients had depression and anxiety associated with life-threatening diseases, namely aggressive oncologic conditions. Although psilocybin is a natural substance found in some mushrooms, it was synthesized (in the laboratory) for the trials and was administered in the form of oral capsules. Concerning the duration of the intervention, results were usually assessed through the scales, days after the administration of psilocybin and in a longer follow-up, in some cases up to three months.

### 3.3. Risk of Publication Bias

**Figure 2:**
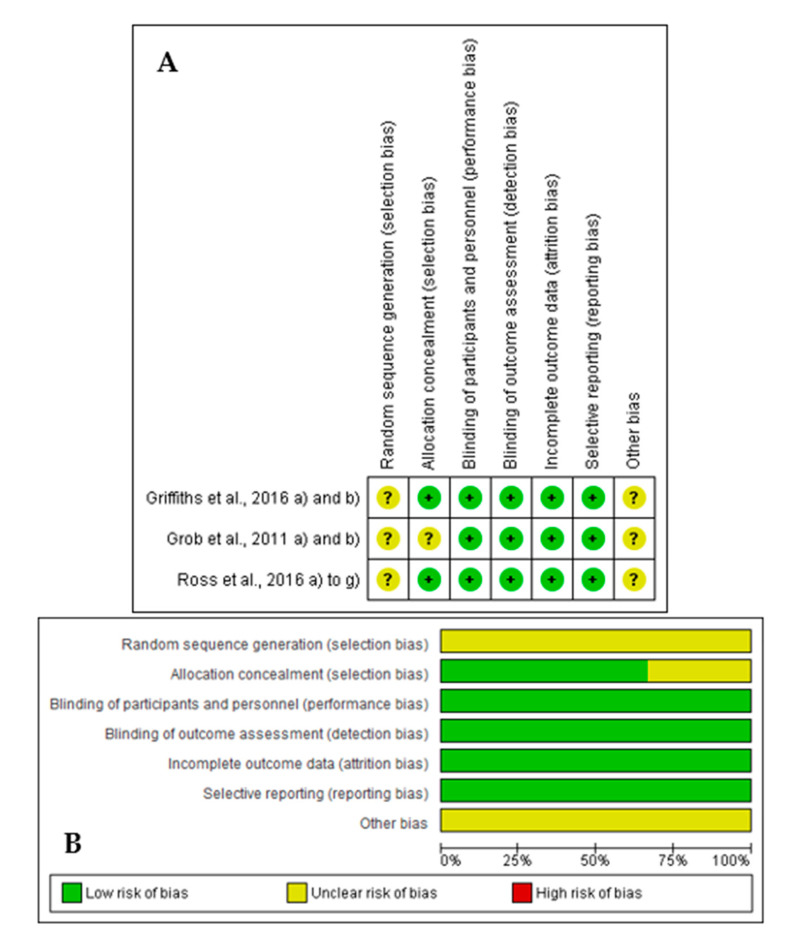
Results of risk of bias assessment regarding the methodological quality of included studies ((**A**) Risk of bias summary: review authors’ judgments about each risk of bias item for each included study; (**B**) Risk of bias graph: review authors’ judgments about each risk of bias item presented as percentages across all included studies).

The results found in the assessment of the risk of publication bias from the included studies are summarized in Figure 2. In general, all the studies satisfied the seven domains of bias defined by Cochrane Collaboration. All the included articles had a focused issue and all the patients who entered the trial were properly accounted for at its conclusion. Aside from the experimental intervention, both the control and intervention groups were treated equally, and all clinically important outcomes were considered. The randomization process was applied in the three articles that were also double-blinded.

### 3.4. Primary Outcomes: Effects of Psilocybin on Depression and Anxiety

BDI and STAI were considered the psychometric scales for this work since they are the most widely used in clinical settings to quantify symptoms of either depression or anxiety.

**Figure 3:**
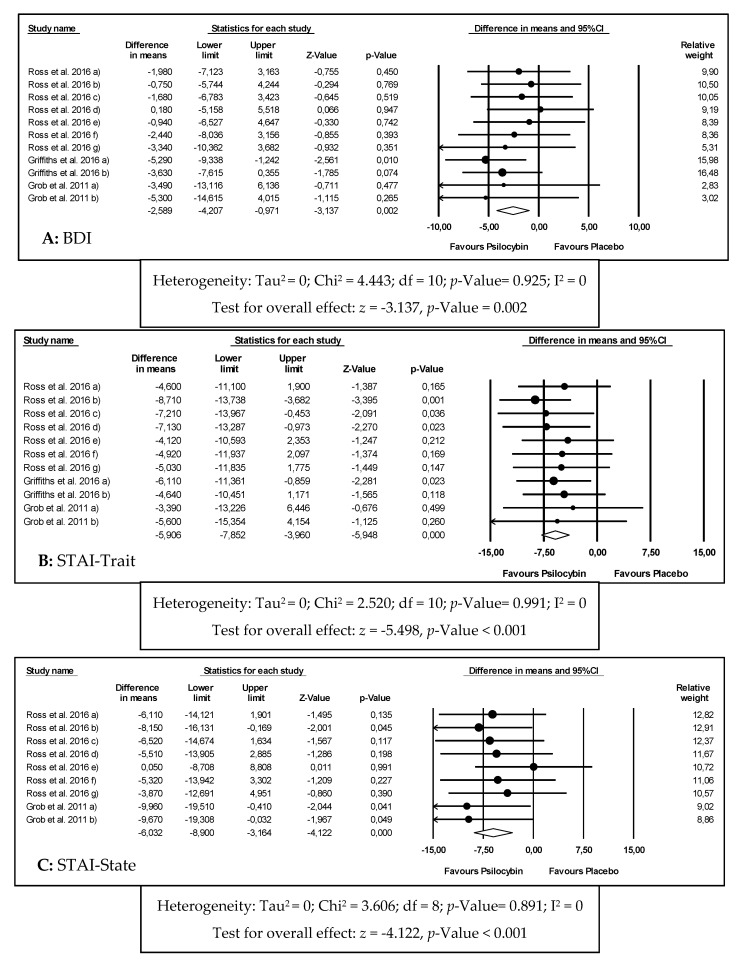
Forest plots of comparisons of the effects of psilocybin on the primary outcomes of this meta-analysis ((**A**) Beck Depression Inventory (BDI); (**B**) State-Trait Anxiety Inventory (STAI)-Trait; (**C**) STAI-State).

The meta-analysis results for the effects of psilocybin in depression through BDI are graphically reported in Figure 3A and Table 2. For BDI, 11 effect sizes were considered, including 92 patients, with a diagnosis of depression and anxiety associated with a life-threatening disease. It was concluded that the intervention group was significantly favored when compared to the control group (WMD = −4.589; 95% CI = −4.207 to −0.971; *p*-value = 0.002). For these results, a fixed effects model was used, given the homogeneity of the studies (I^2^ = 0%).

The meta-analysis results for the effects of psilocybin in anxiety through STAI-Trait and STAI-State are graphically reported in Figure 3B,C, respectively and Table 2.

For STAI-Trait, 11 effect sizes were considered, including 92 patients, with a diagnosis of depression and anxiety associated with a life-threatening disease. Among these, 28 patients had genitourinary cancer, 26 had breast cancer, 16 had digestive cancer, 12 had hematologic malignancies and 10 had other oncologic pathologies. All patients were in advanced stages of their illnesses and some had recurrent metastatic diseases. Apart from that, all patients had also been diagnosed using the DSM V meeting criteria for either chronic adjustment disorder with anxiety, chronic adjustment disorder with mixed anxiety and depressed mood, dysthymic disorder, GAD, MDD or dual diagnosis between GAD and MDD or GAD and dysthymic disorder.

It was concluded that the intervention group was significantly favored when compared to the control group (WMD = −5.906; 95% CI = −7.852 to −3.960; *p*-value ˂ 0.001). For these results, a fixed effects model was used, given the homogeneity between studies (I^2^ = 0%).

For STAI-State, 9 effect sizes were considered, including 41 patients, with a diagnosis of depression and anxiety associated with a life-threatening disease (oncologic conditions). For this outcome, it was concluded that the intervention group was significantly favored when compared to the control group (WMD = −6.032; 95% CI = −8.900 to −3.164; *p*-value ˂ 0.001). For these results, a fixed effects model was used, given the homogeneity between studies (I^2^ = 0%).

### 3.5. Subgroup and Sensitivity Analyses

A subgroup analysis was performed for each primary outcome of the study, except for STAI-State because of the limited number of studies reporting this outcome (Table 3). Accordingly, the influence of psilocybin in either BDI or STAI-Trait was studied separately depending on the dose and on the follow-up time after psilocybin administration.

It was shown that psilocybin induces reduction in both BDI and STAI-Trait at all the tested doses, but the reduction is not dose-dependent. In fact, compared to the control group, this outcome was only statistically significant at the doses of 0.4 mg/kg for BDI and of 0.3 and 0.4 mg/kg for STAI-Trait.

Concerning the time of the follow-up, psilocybin induces statistically significant results in a period of 38 to 189 days in BDI and in 14 to 189 days in STAI-Trait.

The sensitivity analysis was performed by excluding some studies and evaluating how those studies would affect the results (results not shown). This analysis indicates that the pooled effects of psilocybin in depression and anxiety through BDI, STAI-Trait and STAI-State did not change substantially if a few studies were omitted. The sensitivity analysis proved that the overall results obtained in this meta-analysis are robust.

### 3.6. Publication Bias

Publication bias was examined through funnel plots and statistically using the Trim and Fill method (results not shown). Publication bias evaluation was performed separately considering the 3 scales used to measure the effects of psilocybin in depression, through BDI and anxiety, through STAI. Funnel plots indicate asymmetries in the distribution of studies based on sample sizes. The presence of publication bias was further explored using Egger’s regression test. This test indicates evidence of publication bias for the effects of psilocybin on depression and anxiety (*p*-value > 0.05).

### 3.7. Secondary Outcomes: Effects of Psilocybin on Systolic Blood Pressure, Diastolic Blood Pressure and Heart Rate

Systolic blood pressure (SBP), diastolic blood pressure (DBP) and heart rate were evaluated in this meta-analysis as secondary outcomes. The obtained results are summarized in Table 4. As noted, psilocybin produced significant increases in SBP and DBP, up to 6 (*p*-value < 0.017) and 5 h (*p*-value < 0.001) following administration, respectively. The DBP verified increases between 1.194 and 11.381 mmHg, being the average of the increase 7.741 mmHg. Both SBD and DBP tended to stabilize to normal values after 6 or more hours after administration. Psilocybin also significantly increases the heart rate, this increase being highest on the 3rd and 4th hours following administration. Similarly to what was verified for SBP and DBP, heart rate tended to stabilize after 6 or more hours after the administration.

## 4. Discussion

Psilocybin is a molecule with structural similarities to serotonin, an endogenous neurotransmitter. It can be found in some mushrooms and has been widely used to induce hallucinations and altered states of consciousness. Its laboratory synthesis was performed by Albert Hofmann while working at Sandoz Laboratories and it was marketed later under the commercial name Indocybin^®^ for basic psychopharmacology and clinical research [25].

Nonetheless, it was withdrawn in the early 1970s and was classified as a Schedule I drug due to its use outside of medical research and in association with emerging counterculture. Despite withdrawal and criminalization, its potential therapeutic value led many scientists to study the effects and mechanisms of action of the drug. There is preliminary evidence that psilocybin may be useful in the treatment of anxiety and depression in life-threatening diseases, depression, obsessive-compulsive disorder, alcoholism and nicotine addiction, cluster headaches and autism [25]. Although further research is needed to assess the efficacy and safety in the treatment of these conditions, it should be noted that psilocybin is illegal in most countries. Therefore, the illegal status of psilocybin adds complexity and some costs to clinical trials involving its administration to human subjects.

The mechanism of action of psilocybin in depression is still unknown. However, some research suggests that its therapeutic effects in depression may reflect the deactivation of the medial prefrontal cortex (mPFC) that is usually hyperactive in depressed patients [26].

The deactivation of mPFC by psilocybin is detected using functional magnetic resonance imaging (fMRI) and it is correlated with the subjective effects induced by the drug. Other studies with fMRI support that psilocybin attenuates amygdala activation on response to threat-related visual stimuli [27]. This may be one of the mechanisms underlying the therapeutic effectiveness of psilocybin in depression and anxiety, given that the amygdala is extremely important in perception and generation of emotions and amygdala hyperactivity in response to negative stimuli is correlated to negative mood states in depressed patients [28]. Furthermore, the mechanism of action of psilocybin, an agonist of 5-HT(2A) receptors, indicates other evidence of its action in depression, since cortical 5-HT(2A) receptor expression is usually increased in postmortem samples of depressed and suicidal patients [29].

Patients with a potentially life-threatening disease often experience considerable anxiety and psychological distress, depression, anger, loss of perceived self-worth, social isolation, hopelessness, and helplessness [30]. There is no FDA approved pharmacotherapy for psychological distress related with life-threatening diseases and conventional therapy often shows limited efficacy to address symptoms of anxiety and depression. The obtained results in the present work emphasize the importance of psilocybin translational research, when used in the correct environment and with trained professionals and may give clues to future clinical trials.

Psilocybin produces sustained reduction in symptoms of both depression and anxiety. However, a recommendation for its use could only be given under rigorous definition of the conditions and precautions. The efficacy of psychedelic therapy may, unlike conventional pharmacotherapy, be linked to an experiential and meaningful process, responsible for the long-term effects and positive effects in cognition, affect, behavior and spirituality [31]. After psilocybin administration, patients report alleviation from anxiety, reconciliation with death, emotional uncoupling from cancer, spiritual or religious phenomena, reconnection to life and greater confidence [32]. The activation of serotonin receptors 5-HT(2A) mediates perception, attention, and emotional regulation, influencing the waking consciousness, sensory experiences, affectivity, experience of self and dream-like visual imagery [31]. Psilocybin is, therefore, responsible for inducing a profound shift in consciousness, with intensification of affective responses, enhanced ability for introspection, regression to primitive and childlike thinking and activation of vivid memory traces with pronounced emotional processes [33].

The persistence in time of the positive effects of psilocybin suggests that the acute destabilization of brain networks induced by the drug modifies activity in a long-lasting way [34]. Psilocybin may alter acutely brain network activity, decreasing connectivity within the default mode network (DMN) [26]. DMN has a role in consciousness and high-level constructs, such as the self, being essential for the maintenance of cognitive integration and constraint under normal conditions [35].

The increases of both SBP and DBP and heart rate are consistent with the sympathetic effects of psilocybin reported in the literature [36]. The sympathetic effects may also be noticeable through the induction of pupil dilatation [37]. Psilocybin, however, is not likely to cause changes on electrocardiograph or body temperature nor to affect the ionic balance, blood glucose or cholesterol [31].

There were not reported serious adverse effects following psilocybin administration. Besides transient moderate increases in SBD, DBP and heart rate, there are some references to nausea, both physical and psychological discomfort, transient episodes of psychological distress and anxiety. There are, however, no cases of hallucinogen persisting perception disorder (HPPD) neither prolonged psychosis, although literature points these effects to be quite dangerous and life-impairing following hallucinogen consumption [38]. This may occur because in all studies included in the present meta-analysis, patients were carefully monitored in a calm and relaxed environment and had been previously informed about the effects that the drug might have in their bodies and mind. This is known as “set and setting” and it is designed to facilitate a mystical experience and to increase the probability of a positive outcome after the administration of any hallucinogen [25]. In fact, many drug-related and not drug-related variables may influence the adverse effects experienced by patients, namely, age, gender, education, experimental setting, and drug dose [39].

According to the literature, besides the mentioned adverse effects, psilocybin may also cause somatic symptoms such as dizziness, weakness, tremor, drowsiness, yawning, paresthesia, blurred vision, and increased tendon reflexes [40]. Although not applicable to the studies included in this meta-analysis, psilocybin is very likely to induce nausea when consumed through psilocybin-containing mushrooms [41].

Classical hallucinogens are not likely to cause addiction, as it is mainly linked to the dopaminergic system, while classical hallucinogens act mostly on the serotoninergic system. Furthermore, these drugs lead to tachyphylaxis, the rapid decrease in the effect of a drug in consecutive doses, related to their mechanism of action. Thus, the development of addiction by patients after treatment with psilocybin is not of concern. However, authors suggest that if a psilocybin-containing medicines is approved, it should be included in the Schedule IV of Controlled Substance Schedules [42].

## 5. Conclusions

This work demonstrated that psilocybin may be effective in reducing symptoms of depression and anxiety. The results also showed the presence of publication bias, which, however, do not invalidate the conclusions of this meta-analysis. The obtained results are promising and emphasize the importance of psilocybin translational research that may lead to clinically relevant studies. Mechanistic studies are also needed to clarify the mechanism of action of the drug.

## Figures and Tables

**Table 1:** Characteristics of the included studies in this systematic review with meta-analysis.

Study/Year	Sample Size	Study Design	Medical Condition	Mean/Range Age (Years)	Gender (F/M)	Intervention Drug	Control Group	Duration ^1^		Primary Outcomes	Secondary Outcomes
Ross et al., 2016 [19]	29	Double-blind, randomized	Depression and anxiety associated with a life-threatening disease (cancer)	56.28	18/11	Psilocybin, 0.3 mg/kg	Niacin, 250 mg	(a)	1 day post-dose 1	BDI, STAI-State, STAI-Trait	-
								(b)	14 days post-dose 1		
								(c)	42 days post-dose 1		
								(d)	49 days post-dose 1		
								(e)	1 day post-dose 2		
								(f)	42 days post-dose 2		
								(g)	189 days post-dose 2		
Griffiths et al., 2016 [6]	51	Double-blind, randomized	Depression and anxiety associated with a life-threatening disease (cancer)	56.3	25/26	Psilocybin, 0.3 or 0.4 mg/kg	Psilocybin, 1 or 3 mg/70 kg	(a)	28 days pre-crossover	BDI, STAI-Trait	SBP, DBP, HR
									35 days pre-crossover		
								(b)	28 days pre-crossover		
									35 days post-crossover		
Grob et al., 2011 [20]	12	Pilot	Anxiety associated with a life-threatening disease (advanced-stage cancer)	From 36 to 58	11/1	Psilocybin, 0.2 mg/kg	Niacin, 250 mg	(a)	1 day	BDI, STAI-State, STAI-Trait	SBP, DBP, HR
								(b)	14 days		

^1^ The letters in unpaired parenthesis indicate the division of each study in several effect sizes.

**Table 2:** Effects of psilocybin on depression and anxiety.

Outcome Analyzed	Number of Effect Sizes	WMD Observed (95% CI)	*p*-Value	I^2^ (%)	Model USED	WMD Adjusted
						(95% CI)
BDI	11	−4.589	0.002 *	0	Fixed	−4.589
		(−4.207 to −0.971)				(−4.207 to −0.971)
STAI-Trait	11	−5.906	<0.001 *	0	Fixed	−6.389
		(−7.852 to −3.960)				(−8.151 to −4.626)
STAI-State	9	−6.032	<0.001 *	0	Fixed	−6.032
		(−8.900 to −3.164)				(−8.900 to −3.164)

WMD—weighted mean differences; CI—confidence interval; * Indicates a significant result.

**Table 3:** Subgroup analysis of the effects of psilocybin on depression and anxiety.

**Variable**	**BDI**				**STAI−Trait**			
	**Number of Effect Sizes**	**95% CI**	***p*−Value**	**I^2^ (%)**	**Number of Effect Sizes**	**95% CI**	***p*−Value**	**I^2^ (%)**
Total	11	−	-	-	11	−	−	−
WMD observed	-	−2.589	0.002 *	0	-	−5.906	<0.001 *	0
		(−4.207 to −0.971)				(−7.852 to −3.960)		
WMD adjusted	-	−2.589	-	-	-	−5.906	-	-
		(−4.207 to −0.971)				(−7.852 to −3.960)		
**Dose (mg/kg)**	**BDI**				**STAI−Trait**			
	**Number of Effect Sizes**	**95% CI**	***p*−Value**	**I^2^ (%)**	**Number of Effect Sizes**	**95% CI**	***p*−Value**	**I^2^ (%)**
0.2	2	−4.425	0.195	0	2	−4.509	0.208	0
		(−11.118 to −2.269)				(−11.430 to 2.422)		
0.3	7	−1.438	0.171	0	7	−6.240	<0.001 *	0
		(−3.498 to 0.622)				(−8.615 to −3.865)		
0.4	2	−4.447	0.002 *	0	2	−5.449	0.008 *	0
		(−7.287 to −1.607)				(−9.345 to −1.553)		
**Time (days) after Psilocybin Administration**	**BDI**				**STAI−Trait**			
	**Number of Effect Sizes**	**95% CI**	***p*−Value**	**I^2^ (%)**	**Number of Effect Sizes**	**95% CI**	***p*−Value**	**I^2^ (%)**
1	3	−1.769	0.325	0	2	−4.359	0.067	0
		(−5.290 to 1.752)				(−8.946 to 0.228)		
14	2	−1.766	0.432	0	3	−7.258	0.005 *	39.340
		(−6.167 to 2.635)				(−11.330 to −3.184)		
35–189	6	−3.024	0.003 *	0	6	−5.854	<0.001 *	0
		(−5.026 to −1.023)				(−8.385 to −3.322)		

WMD—weighted mean differences; CI—confidence interval; * Indicates a significant result.

**Table 4:** Meta-analysis for secondary outcomes.

Time (Hours) after Psilocybin Administration	WMD (95% CI)	*p*-Value	I^2^ (%)
**Systolic blood pressure (SBP)**			
1	13.824 (9.433 to 18.216)	<0.001 *	0
2	22.601 (18.075 to 27.128)	<0.001 *	0
3	18.939 (14.893 to 22.986)	<0.001 *	49.577
4	15.252 (10.998 to 19.505)	<0.001 *	0
5	7.978 (4.184 to 11.773)	<0.001 *	0
6	4.222 (0.792 to 7.652)	0.016 *	0
Overall	12.741 (11.100 to 14.382)	<0.001 *	82.280
**Diastolic blood pressure (DBP)**			
1	11.242 (8.843 to 13.640)	<0.001 *	0
2	11.020 (8.598 to 13.441)	<0.001 *	61.227
3	11.381 (9.020 13.743)	<0.001 *	52.635
4	8.625 (6.080 to 11.170)	<0.001 *	0
5	4.912 (2.374 to 7.450)	<0.001 *	0
6	1.194 (-0.883 to 3.271)	0.260	0
Overall	7.741 (6.722 to 8.710)	<0.001 *	86.350
**Heart Rate**			
1	5.056 (0.787 to 9.325)	0.020 *	0
2	8.268 (4.115 to 12.421)	<0.001 *	0
3	8.281 (4.288 to 12.275)	<0.001 *	44.279
4	8.520 (4.495 to 12.546)	<0.001 *	64.899
5	5.599 (1.495 to 9.703)	0.008 *	0
6	3.363 (-0.716 to 7.441)	0.106	13.130
Overall	6.549 (4.875 to 8.223)	<0.001 *	24.596

WMD—weighted mean differences; CI—confidence interval; * Indicates a significant result.

## References

[1] Akil H., Gordon J., Hen R., Javitch J., Mayberg H., McEwen B., Meaney M.J., Nestler E.J. (2018). Treatment resistant depression: A multi-scale, systems biology approach. Neurosci. Biobehav. Rev..

[2] McIntyre R.S., Filteau M., Martin L., Patry S., Carvalho A., Cha D.S., Barakat M., Miguelez M. (2014). Treatment-resistant depression: Definitions, review of the evidence, and algorithmic approach. J. Affect. Disord..

[3] Passik S.D., Kirsh K.L., Rosenfield B., McDonald M.V., Theobald D.E. (2001). The changeable nature of patients’ fears regarding chemotherapy: Implications for palliative care. J. Pain Symptom Manag..

[4] Groenvold M., Petersen M.A., Idler E., Bjorner J.B., Fayers P.M., Mouridsen H.T. (2007). Psychological distress and fatigue predicted recurrence and survival in primary breast cancer patients. Breast Cancer Res. Treat..

[5] Pinquart M., Duberstein P.R. (2010). Depression and cancer mortality: A meta-analysis. Psychol. Med..

[6] Griffiths R.R., Johnson M.W., Carducci M.A., Umbricht A., Richards W.A., Richards B.D., Cosimano M.P., Klinedinst M.A. (2016). Psilocybin produces substantial and sustained decreases in depression and anxiety in patients with life-threatening cancer: A randomized double-blind trial. J. Psychopharmacol..

[7] Grassi L., Spiegel D., Riba M. (2017). Advancing psychosocial care in cancer patients. F1000Research.

[8] Freedman R.A., Kouri E.M., West D.W., Lii J., Keating N.L. (2016). Association of Breast Cancer Knowledge With Receipt of Guideline-Recommended Breast Cancer Treatment. J. Oncol. Pract..

[9] Orhurhu V.J., Claus L.E., Cohen S.P. (2019). Ketamine Toxicity.

[10] Goldberg S.B., Pace B.T., Nicholas C.R., Raison C.L., Hutson P.R. (2020). The experimental effects of psilocybin on symptoms of anxiety and depression: A meta-analysis. Psychiatry Res..

[11] Moher D., Shamseer L., Clarke M., Ghersi D., Liberati A., Petticrew M., Shekelle P., Stewart L.A. (2015). Preferred Reporting Items for Systematic Review and Meta-Analysis Protocols (PRISMA-P) 2015 statement. Syst. Rev..

[12] Moher D., Liberati A., Tetzlaff J., Altman D.G. (2009). Preferred Reporting Items for Systematic Reviews and Meta-Analyses: The PRISMA Statement. Ann. Intern. Med..

[13] Beck A.T., Ward C.H., Mendelson M., Mock J., Erbaugh J. (1961). An inventory for measuring depression. Arch. Gen. Psychiatry.

[14] Julian L.J. (2011). Measures of anxiety: State-Trait Anxiety Inventory (STAI), Beck Anxiety Inventory (BAI), and Hospital Anxiety and Depression Scale-Anxiety (HADS-A). Arthritis Care Res..

[15] Ryan R., Hill S., Prictor M., McKenzie J. (2013). Cochrane Consumers & Communication Review Group Study Quality Guide Guide for Review Authors on Assessing Study Quality. https://cccrg.cochrane.org/sites/cccrg.cochrane.org/files/public/uploads/StudyQualityGuide_May%202013.pdf.

[16] Higgins J.P.T., Altman D.G., Gotzsche P.C., Juni P., Moher D., Oxman A.D., Savocic J., Schilz K.F., Weeks L., Sterne J.A.C. (2011). The Cochrane Collaboration’s tool for assessing risk of bias in randomised trials. BMJ.

[17] Borenstein M., Hedges L., Higgins J. (2009). Introduction to Meta-Analysis.

[18] Higgins J., Thompson S.G., Deeks J.J., Altman D.G. (2003). Measuring inconsistency in meta-analyses. BMJ.

[19] Ross S., Bossis A., Guss J., Agin-Liebes G., Malone T., Cohen B., Mennenga S.E., Belser A., Kalliontzi K., Babb J. (2016). Rapid and sustained symptom reduction following psilocybin treatment for anxiety and depression in patients with life-threatening cancer: A randomized controlled trial. J. Psychopharmacol..

[20] Grob C.S., Danforth A.L., Chopra G.S., Hagerty M., McKay C.R., Halberstadt A.L., Greer G.R. (2011). Pilot Study of Psilocybin Treatment for Anxiety in Patients With Advanced-Stage Cancer. Arch. Gen. Psychiatry.

[21] Stroud J.B., Freeman T.P., Leech R., Hindocha C., Lawn W., Nutt D.J., Curran H.V., Carhart-Harris R.L. (2018). Psilocybin with psychological support improves emotional face recognition in treatment-resistant depression. Psychopharmacology.

[22] Lyons T., Carhart-Harris R.L. (2018). More Realistic Forecasting of Future Life Events After Psilocybin for Treatment-Resistant Depression. Front. Psychol..

[23] Carhart-Harris R.L., Bolstridge M., Day C.M.J., Rucker J., Watts R., Erritzoe D.E., Kaelen M., Giribaldi B., Bloomfield M., Pillimg S. (2018). Psilocybin with psychological support for treatment-resistant depression: Six-month follow-up. Psychopharmacology.

[24] Carhart-Harris R.L., Bolstridge M., Rucker J., Day C.M.J., Erritzoe D., Kaelen M., Bloomfield M., Rickard J.A., Forbes B., Feilding A. (2016). Psilocybin with psychological support for treatment-resistant depression: An open-label feasibility study. Lancet.

[25] Nichols D.E. (2016). Psychedelics. Pharmacol. Rev..

[26] Carhart-Harris R.L., Erritzoe D., Williams T., Stone J.M., Reed L.J., Colasanti A., Tyacke R.J., Leech R., Malizia A.L., Murphy K. (2012). Neural correlates of the psychedelic state as determined by fMRI studies with psilocybin. Proc. Natl. Acad. Sci. USA.

[27] Kraehenmann R., Preller K.H., Scheidegger M., Pokorny T., Bosch O.G., Seifritz E., Vollenweider F.X. (2015). Psilocybin-Induced Decrease in Amygdala Reactivity Correlates with Enhanced Positive Mood in Healthy Volunteers. Biol. Psychiatry.

[28] DeRubeis R.J., Siegle G.J., Hollon S.D. (2008). Cognitive therapy versus medication for depression: Treatment outcomes and neural mechanisms. Nat. Rev. Neurosci..

[29] Pandey G.N., Dwivedi Y., Rizavi H.S., Ren X., Pandey S.C., Pesold C., Roberts R.C., Conley R.R., Tamminga C.A. (2002). Higher expression of serotonin 5-HT(2A) receptors in the postmortem brains of teenage suicide victims. Am. J. Psychiatry.

[30] Breitbart W., Rosenfeld B., Pessin H., Kaim M., Funesti-Esch J., Galietta M., Nelson C.J., Brescia R. (2000). Depression, Hopelessness, and Desire for Hastened Death in Terminally Ill Patients With Cancer. JAMA.

[31] Hasler F., Grimberg U., Benz M.A., Huber T., Vollenweider F.X. (2004). Acute psychological and physiological affects of psilocybin in healthy humans: A double-blind, placebo-controlled dose-effect study. Psychopharmacology.

[32] Swift T.C., Belser A.B., Agin-Liebes G., Devenot N., Terrana S., Friedman H.L., Guss J., Bossis A.P., Ross S. (2017). Cancer at the Dinner Table: Experiences of Psilocybin-Assisted Psychotherapy for the Treatment of Cancer-Related Distress. J. Humanist. Psychol..

[33] Studerus E., Kometer M., Hasler F., Vollenweider F.X. (2011). Acute, subacute and long-term subjective effects of psilocybin in healthy humans: A pooled analysis of experimental studies. J. Psychopharmacol..

[34] Nichols D.E., Johnson M.W., Nichols C.D. (2017). Psychedelics as Medicines: An Emerging New Paradigm. Clin. Pharmacol. Ther..

[35] Raichle M.E. (1998). The neural correlates of consciousness: An analysis of cognitive skill learning. Philos. Trans. R. Soc. Lond. B. Biol. Sci..

[36] Passie T., Seifert J., Schneider U., Emrick H.M. (2002). The pharmacology of psilocybin. Addict. Biol..

[37] Isbell H. (1959). Comparison of the reactions induced by psilocybin and LSD-25 in man. Psychopharmacologia.

[38] Hermle L., Simon M., Ruchsow M., Geppert M. (2012). Hallucinogen-persisting perception disorder. Ther. Adv. Psychopharmacol..

[39] Studerus E., Gamma A., Kometer M., Vollenweider F.X. (2012). Prediction of psilocybin response in healthy volunteers. PLoS ONE.

[40] Johnson M., Richards W., Griffiths R. (2008). Human hallucinogen research: Guidelines for safety. J. Psychopharmacol..

[41] Tylš F., Páleníček T., Horáček J. (2014). Psilocybin—Summary of knowledge and new perspectives. Eur. Neuropsychopharmacol..

[42] Johnson M.W.M., Griffiths R.R., Hendricks P.S., Henningfield J.E. (2018). The abuse potential of medical psilocybin according to the 8 factors of the Controlled Substances Act. Neropharmacology.

